# Beyond visual inspection: the deep learning revolution in quantitative cerebrovascular imaging

**DOI:** 10.3389/fnins.2025.1768107

**Published:** 2026-07-16

**Authors:** Xuewei Mao, Huajun Yang, Shiwen Weng, Fangyun Peng, Jiazhen Xu

**Affiliations:** 1 School of Automation and the Shandong Key Laboratory of Industrial Control Technology, Qingdao University, Qingdao, China; 2 Neurological Department, Sanbo Brain Hospital, Capital Medical University, Beijing, China; 3 Department of Neurology, Beijing Tiantan Hospital, Capital Medical University, Beijing, China; 4 Department of Pharmacology, School of Pharmacy, Qingdao University, Qingdao, Shandong, China

**Keywords:** cerebrovascular imaging, deep learning, image segmentation, quantitative imaging, stroke diagnosis

## Abstract

The rising global burden of cerebrovascular disease, propelled by an aging population, highlights the inherent limitations of conventional, labor-intensive diagnostic paradigms. In the context of time-sensitive stroke management, variability in image interpretation and the high rate of misclassification, particularly during the assessment of transient ischemic attack (TIA), underscore the urgent need for more consistent and efficient diagnostic solutions. Artificial intelligence (AI), particularly deep learning (DL), offers a transformative pathway by automating the analysis of complex neurovascular imaging. Here, we conduct a comprehensive examination of how DL is revolutionizing stroke-related image analysis, moving beyond general assertions of potential to discuss specific technical implementations. We systematically detail the evolution from traditional segmentation algorithms to advanced deep learning architectures—such as U-Net, DeepMedic, and their variants—in performing critical tasks. These tasks encompass the automated segmentation of intracranial and extracranial (carotid) arteries, the quantification of stenosis and plaque burden, and the hemodynamic assessment of vascular lesions across modalities including MRA, CTA, and DSA. By synthesizing landmark studies, our analysis delineates three core aspects: the technological trajectory of DL models in achieving expert-level accuracy in vascular feature extraction in controlled studies; the clinical translation of these tools into diagnostic, prognostic, or therapeutic procedural planning workflows; and the persistent challenges and future directions, including data standardization, model generalizability, and multimodal integration. This review posits that DL represents not merely an assistive technology but a foundational cornerstone for the next generation of precision cerebrovascular medicine. It holds the potential to bridge critical gaps in diagnostic speed, objectivity, and accessibility, provided its development and validation are guided by rigorous, interdisciplinary collaboration.

## 1 Introduction

Stroke remains the second leading cause of death globally, accounting for 11.6% of total mortality (Chen et al., 2025) and imposing projected annual economic costs exceeding $2.3 trillion by 2030 (Bennitt et al., 2025). This pressing public health challenge reveals fundamental limitations in conventional diagnostic paradigms that rely heavily on subjective visual assessment of neuroimaging. Current clinical practice is constrained by three major bottlenecks. First, the critical time-sensitive diagnostic window is often compromised by limited human resources and interpreter fatigue, with studies documenting significant variability in imaging interpretation even among specialists (Chu et al., 2022). Second, the evaluation of subtle imaging findings—such as those associated with transient ischemic attacks or early-stage stenosis—is highly susceptible to error, and misclassification at initial assessment can lead to disabling, preventable secondary strokes. Finally, the rapid expansion of multimodal neuroimaging data (e.g., MRA, CTA, DSA) has outpaced the capacity for manual analysis. This results in workflow delays that hinder the precise quantitative assessment of vascular morphology, plaque burden, and hemodynamics—parameters essential for tailored, personalized treatment planning.

The emergence of artificial intelligence (AI), particularly deep learning (DL), in medical imaging represents a paradigm shift with the potential to transcend rapid detection and enable comprehensive vascular analysis. Deep learning algorithms—notably convolutional neural networks and U-Net-based architectures—have evolved to automate the core tasks of stroke diagnosis, from segmenting intricate intracranial and extracranial vasculature to quantifying stenosis, plaque volume, and key hemodynamic parameters. These systems can process complex, multi-dimensional imaging data in seconds, thereby performing detailed analyses that are prohibitively time-consuming manually, all while demonstrating diagnostic performance comparable to that of vascular neuroradiology experts in controlled studies (Kousar et al., 2025). This advancement promises to address the critical triad of challenges in cerebrovascular assessment: speed, consistency, and depth.

This review synthesizes evidence from landmark and contemporary studies to critically analyze the trajectory of AI in stroke neuroimaging. We structure our discussion across three pivotal dimensions: first, tracing the technological evolution from basic machine learning (ML) to advanced deep learning models for vascular image analysis; second, evaluating the clinical translation of these tools, including validation studies, implementation barriers, and emerging applications in both ischemic and hemorrhagic stroke; and finally, proposing a forward-looking strategic roadmap that outlines the prerequisites for sustainable clinical integration. Our analysis aims to bridge the gap between computational innovation and bedside practice, thereby aiming to serve as both a technical primer for developers and a practical roadmap for healthcare systems embarking on the integration of AI into the stroke care pathway.

## 2 The historical foundations and core paradigms of artificial intelligence

Artificial intelligence (AI) has evolved significantly from its early rule-based expert systems to modern data-driven approaches, revolutionizing fields like medical imaging. A pivotal shift occurred with the rise of machine learning (ML), which enables systems to learn patterns directly from data (Lusted, 1955). This was accelerated by deep learning (DL), a powerful subset of ML that uses multi-layered neural networks for complex pattern recognition. Recent progress in perceptual AI has markedly enhanced the interpretation of multimodal data, with medical imaging being a primary beneficiary (Yang J. et al., 2025).

In medical imaging, DL provides two key capabilities: automated feature extraction and advanced interpretation, establishing correlations between imaging biomarkers and clinical endpoints. These techniques leverage information often imperceptible to the human eye, redefining precision medicine paradigms.

Deep learning architectures are broadly categorized by training data. Supervised networks learn from labeled datasets for tasks like classification, while unsupervised networks identify latent patterns in unlabeled data. Predominant frameworks include Convolutional Neural Networks (CNNs) for spatial feature extraction in 2D/3D images, Recurrent Neural Networks (RNNs) for sequential data, and generative models like GANs and VAEs for data augmentation and representation learning (Scalco et al., 2025; Shobayo and Saatchi, 2023). The power of DL lies in its ability to capture subtle, clinically relevant patterns within radiological images, enhancing diagnostic accuracy and clinical decision-making (Hosny et al., 2018).

## 3 Data infrastructure and initial applications of deep learning in radiology

The establishment of Picture Archiving and Communication Systems (PACS) has facilitated the archival and management of large-scale medical imaging datasets, which are essential for training deep learning (DL) models. Related studies have also demonstrated the value of imaging-based assessment, radiomics, and machine learning or deep learning methods in carotid stenosis management, vascular risk stratification, edema quantification, and lesion characterization across different clinical imaging contexts (Dharmakidari et al., 2017; Hua et al., 2015; Jeong et al., 2024; Skoda et al., 2023; Wang et al., 2017; Chen et al., 2016; Zhuge et al., 2020; Coroller et al., 2015). While DL has advanced numerous radiological applications, its impact on vascular imaging—particularly in the coronary domain—has been substantial. Research on vascular segmentation has extensively focused on both coronary and intracranial arteries, with many studies demonstrating high accuracy and robustness (Lenfant et al., 2022; Liu et al., 2024).

In coronary artery imaging, DL techniques have been successfully applied to tasks such as automated vessel segmentation, detection and grading of luminal stenosis, and quantitative assessment of coronary artery calcium (CAC). For instance, a model developed by Paul et al. achieved 96% accuracy in detecting significant stenosis (Paul et al., 2022). Deep learning has also addressed limitations in conventional CAC scoring by enabling quantification directly from contrast-enhanced scans, thereby reducing scan time and radiation exposure (Mu et al., 2022). Furthermore, DL-based approaches have emerged as promising solutions for computationally intensive tasks such as non-invasive fractional flow reserve (FFR) estimation. Li et al. developed a dual-sampling channel network that reduced computation time by 600-fold while maintaining generalizability for evaluating complex arterial systems (Li et al., 2021). Beyond computed tomography applications, frameworks incorporating attention mechanisms have been developed for fully automatic stenosis identification in X-ray angiography, improving localization precision and reducing reliance on manual assessment (Moon et al., 2021).

The demonstrated success of DL in the well-established domain of coronary imaging provides a valuable benchmark and a source of methodological insights. However, cerebrovascular imaging presents distinct and often greater challenges—such as more complex vessel tortuosity, smaller vessel diameters, dynamic flow variations in the Circle of Willis, and the need for precise differentiation between ischemic and hemorrhagic substrates. These complexities necessitate tailored architectural adaptations and deeper discussion, to which the reclaimed space in this review is now dedicated. The comparative efficiency of DL in coronary applications thus serves to highlight the unique requirements and unresolved questions in the cerebrovascular domain.

## 4 Core deep learning architectures

The remarkable progress in cerebrovascular image analysis is largely driven by several foundational deep learning architectures. Understanding these core models is essential, as most advancements represent specialized adaptations of these paradigms to address specific medical imaging challenges.

**Convolutional Neural Networks (CNNs)** form the bedrock of modern image analysis. Their hierarchical structure, comprising convolutional, pooling, and fully connected layers, enables automatic learning of spatial features—from simple edges in early layers to complex shapes in deeper layers (Sivonxay et al., 2025). This intrinsic property makes CNNs exceptionally powerful for tasks like image classification and object detection, serving as the fundamental building blocks for more complex architectures discussed below.

The **U-Net** architecture, arguably the most influential model in biomedical image segmentation, addresses a critical need: precise pixel-wise localization (Ronneberger et al., 2015). Its symmetric encoder-decoder structure, connected by “skip connections,” allows the network to simultaneously leverage high-level contextual information from the contracting path and high-resolution spatial details from the expanding path. This design is particularly adept at handling the tubular, branching structures of vasculature, as it prevents the loss of fine details necessary for accurately delineating small vessels. Its success has spawned numerous variants (e.g., 3D U-Net, U-Net++) tailored for volumetric data and improved performance (Sun et al., 2025; Zhou et al., 2018).

For complex 3D volumes, architectures like **DeepMedic** offer a powerful alternative (Vossough et al., 2024). DeepMedic processes input images at multiple parallel scales using dual pathways, enabling the network to capture both fine-grained details and broader contextual information simultaneously. This multi-scale approach is especially beneficial for cerebrovascular applications, where the algorithm must recognize large vessels like the internal carotid artery while also precisely segmenting small, distal branches, all within a 3D space.

More recently, **Transformer-based models**, which utilize self-attention mechanisms, have been adapted from natural language processing to computer vision (Sujit et al., 2025). These models excel at capturing long-range dependencies within an image, potentially modeling complex relationships across different parts of a vascular tree. While computationally demanding, they represent a promising frontier for understanding global anatomical context in vascular imaging (Ganesh and Kannadhasan, 2023).

The subsequent sections will explore how these core architectures—U-Net, DeepMedic, and Transformers—are specifically adapted, combined, and optimized for distinct tasks such as intracranial vs. carotid artery analysis, moving from foundational principles to application-specific innovations.

## 5 Traditional and deep learning approaches for intracranial artery segmentation

Within head and neck vasculature imaging, deep learning (DL) applications have predominantly focused on intracranial arteries. Conventional evaluation of intracranial vasculature predominantly relies on visual, qualitative assessment in clinical practice, whereas the quantitative extraction of cerebral arteries and their morphological parameters is considerably more challenging. Manual or semi-automatic vessel segmentation remains labor-intensive, time-consuming, and subject to inter-observer variability. The central premise of DL-based cerebrovascular segmentation algorithms involves training specialized network architectures on extensively annotated datasets, enabling them to directly map raw image data to accurate segmentation masks for automated vascular feature extraction.

Methods for extracting cerebral vasculature from magnetic resonance angiography (MRA) images can be broadly categorized as skeleton-based or non-skeleton-based. Skeleton-based approaches first extract vessel centerlines to construct a vascular tree, followed by geometric modeling to represent the centerline structure explicitly or implicitly. However, these methods often fail to provide a complete volumetric reconstruction of the vasculature. In contrast, non-skeleton-based approaches aim to directly segment vessels from 3D MRA data, typically using deformable models or thresholding techniques. A prominent example is the level set method, a deformable model that can track vascular boundaries by iteratively minimizing an energy function (Ullah et al., 2023). While numerous improved level set variants have been applied to vascular segmentation, their practical utility is often limited by sensitivity to initialization, computational cost, and convergence behavior.

Regarding thresholding methods, Wang et al. (2015) proposed a technique for segmenting cerebrovascular structures from three-dimensional time-of-flight (TOF)-MRA, a non-contrast technique widely adopted in clinical practice due to its rapid acquisition and high native contrast. Their method applied Otsu's thresholding to partition the image, modeled the foreground voxel intensities using combined normal and Gumbel distributions, and determined the final segmentation threshold by comparing the probability density functions of vascular and tissue distributions. The resulting segmentation achieved a Dice similarity coefficient (DSC) >0.7, demonstrating its potential utility for 3D visualization and quantification. In the realm of deformable models, Zhao et al. (2014) implemented a cerebrovascular segmentation approach using a novel Allen–Cahn equation coupled with a likelihood-based level set algorithm. The Allen–Cahn equation governed curve evolution, while an integrated Gaussian distribution model helped address low-contrast regions to detect finer vascular details. Comparative evaluations against LBF and LCV models indicated that this method achieved superior performance with fewer iterations and reduced computational time.

As contrast-enhanced T1-weighted (T1CE) MRI sequences are integral to stereotactic planning, Neumann et al. (2019) developed a software tool implementing three distinct vascular segmentation algorithms for cerebral vessel detection from T1CE and MRA images. The study combined level set and vessel enhancement filtering techniques and evaluated their performance using three distinct analytical methods: vesselness, fast marching, and level set. Among the three methods evaluated, the fast marching algorithm demonstrated the best performance, whereas the vesselness method yielded substantially inferior results. The level set method performed consistently well across both MPRAGE and 3T-TOF/7T-TOF image sets. Their work demonstrates the feasibility of integrating clinically meaningful vessel detection and collision warning systems into stereotactic planning software within a clinical software environment.

In model-driven cerebrovascular segmentation, maximum a posteriori (MAP) and Markov random field (MRF) frameworks are commonly employed. A key limitation of these statistical models is their lack of robustness when applied to data from MR scanners with varying types and acquisition parameters, which hinders accurate and generalizable vessel segmentation. Zhang et al. (2019) addressed this issue by refining a model-driven segmentation algorithm. First, they applied a regional histogram normalization to TOF-MRA data. Subsequently, a Gaussian mixture model (GMM) with three components was constructed, and its parameters were estimated using the expectation-maximization algorithm. Finally, probability feature maps derived from the GMM's estimated vascular distribution were embedded into an MRF framework to suppress label noise and enhance vascular structures. This approach demonstrated superior accuracy and improved sensitivity for small vessels and large arteriovenous malformations compared to alternative models. Zhou S. et al. (2019) also enhanced the MAP-MRF framework by implementing a GMM in the low-level process for skull stripping and feature extraction from TOF-MRA data, while incorporating a novel pairwise spatial potential function within the Markov neighborhood system (NBS) at the high-level process to model spatial dependencies. They utilized machine learning to automatically estimate the Markov regularization parameters, eliminating the need for empirical, dataset-specific tuning. This model improved segmentation accuracy and reduced vascular discontinuities in regions with low contrast or signal-to-noise ratio.

The fundamental principle of these traditional algorithms is their reliance on hand-crafted rules and heuristics designed to match presumed vascular characteristics. Their primary advantages are conceptual simplicity and computational efficiency. However, such manually defined features have inherently limited generalizability and may struggle to capture the full heterogeneity of vascular appearances. For instance, while simple and fast, threshold-based segmentation is highly susceptible to high-intensity imaging artifacts and noise. Similarly, level set methods can adapt to complex geometries, but their performance remains highly dependent on the careful design of the energy function and initialization.

Deep neural networks (DNNs) can automatically learn and refine vascular features through training, overcoming key limitations of traditional segmentation algorithms and achieving superior performance. Consequently, deep learning-based methods are gradually becoming the mainstream approach for vascular segmentation. This transition is particularly relevant for digital subtraction angiography (DSA), which is regarded as the gold standard for diagnosing cerebrovascular diseases due to its ability to provide high-resolution, high-contrast visualization of vascular anatomy. Automatic and accurate segmentation of cerebrovascular structures from DSA can significantly aid diagnosis and facilitate the reconstruction of 3D vascular models for improved lesion assessment (Banerjee et al., 2024). However, segmenting cerebral vessels in DSA presents distinct challenges. The cerebrovascular tree encompasses a wide range of calibers, from fine capillaries to larger arteries like the internal carotid, necessitating the consideration of multi-scale segmentation targets. Simultaneously, inhomogeneous contrast opacification during DSA imaging can introduce artifacts and noise. These factors make robust automatic segmentation a challenging computational task.

To address these issues, Meng et al. (2020) proposed a multi-scale dense convolutional neural network (MDCNN) framework for DSA image segmentation. Inspired by U-Net, the MDCNN employs an encoder-decoder structure. Its key innovations include a dedicated multi-scale module to handle vessels of varying diameters and redesigned skip connections to better utilize encoder features. An improved dense block was also incorporated to enhance high-level feature extraction. Evaluated on the DCVesser dataset, this framework achieved high performance metrics, including F1-score, accuracy, and AUC, demonstrating the superiority of this approach for DSA segmentation. In another approach, Phellan et al. (2017) employed an 8-layer convolutional autoencoder (CAE) to learn a 3D segmentation model for intracranial arteries. By leveraging the autoencoder's ability to learn low-dimensional representations of high-dimensional data, the model effectively reduced noise and extracted relevant features. After parameter optimization, the trained model demonstrated promising performance in binary classification and visual assessment, with Dice similarity coefficient (DSC) values ranging from 0.764 to 0.786.

While deep neural network (DNN)-based segmentation methods demonstrate performance superior to traditional machine learning techniques, highlighting the potential of deep CNNs for TOF-MRA analysis, their robustness is often constrained by reliance on training data from a single scanner type and resolution. Furthermore, achieving generalizability across diverse devices necessitates vast volumes of manually annotated TOF-MRA images from varied sources. The anatomical complexity of the human cerebrovasculature makes large-scale manual annotation prohibitively expensive. To overcome this scarcity of labeled data, Zhang et al. (2020) proposed a novel, automated cerebrovascular segmentation strategy that integrates model-driven and data-driven approaches. First, sparse annotations were provided by radiologists on TOF-MRA datasets. Second, a semi-supervised hybrid probability model was applied to these sparse labels to precisely fit the cerebrovascular density distribution, generating a large set of candidate labeled points. Third, a consistency-checking model (C-M model) corrected mislabeled points within this set, yielding a high-quality pseudo-label set. These steps significantly reduced the dependency on exhaustive manual annotation. Finally, this refined label set was used to construct and train a Deep Discrete Convolutional Neural Network (DD-CNN), designed to enhance multi-scale spatial feature learning efficiently. A key architectural feature was the use of short connections between feature maps, which alleviated the vanishing gradient problem, facilitated convergence, and aided multi-level feature extraction. The model maintained relatively low computational cost while achieving strong performance, demonstrating improved completeness and sensitivity in segmenting cerebrovascular structures, producing smoother vessel boundaries, effectively suppressing pseudo-vessel artifacts, and allowing clear visualization of vascular malformations.

In TOF-MRA images, cerebrovascular structures share common morphological features, with primary differences residing in intensity contrast and caliber. Since the Hidden Markov Random Field (HMRF) model is effective for segmenting larger, higher-contrast vessels but less sensitive to smaller ones, and deep neural networks (DNNs) excel at learning multi-resolution features through hierarchical structures, a hybrid strategy can be advantageous. A DNN model trained on initial labels generated by an HMRF can achieve stronger performance in identifying small vessels compared to using HMRF alone. This approach reduces reliance on extensive manual annotation and maintains robust performance across TOF-MRA images from different scanners and resolutions.

Fan et al. (2020) proposed a novel unsupervised cerebrovascular segmentation framework combining DNNs with an HMRF model. In their pipeline, the HMRF first provides automated initial segmentation, generating region-of-interest (ROI) labels to train the DNN. A preprocessing step sets background intensity to zero, labeling it as a separate class to improve algorithm performance. The study constructed two architectures: a 2D SegNet and a 3D U-Net, for segmenting the cerebrovascular tree. To enhance the 2D SegNet's performance, an HMRF+SegNet2D model was developed. Leveraging the neurophysiological principle that cerebrovascular systems share similar 3D topological structures across individuals, this model incorporated three sub-SegNet networks, each trained on 2D TOF-MRA images from the axial, sagittal, and coronal planes, respectively. The final probability map was generated by averaging the outputs from these three orthogonal networks. Experimental results demonstrated that both the HMRF+SegNet2D and HMRF+U-Net3D models achieved competitive cerebrovascular segmentation performance, outperforming the HMRF method alone and effectively combining the strengths of both approaches.

While deep convolutional networks have achieved remarkable success in visual recognition tasks, their application in medical imaging is often constrained by the need for large, annotated datasets and the complexity of network design. This challenge is particularly evident in the automatic segmentation of intracranial arteries, where early studies utilizing relatively shallow networks on small datasets yielded limited performance and clinical applicability. The introduction of the U-net architecture by Ronneberger et al. (2015), based on a fully convolutional network (FCN), marked a significant advancement. U-net addressed key issues in medical image segmentation through its symmetric encoder-decoder structure with skip connections, enabling precise localization and context capture. Its ability to perform end-to-end learning without manual feature engineering, coupled with data-efficient training strategies like elastic deformation for augmentation, made it superior to many traditional methods and established it as a dominant architecture in biomedical image segmentation.

Following this, Livne et al. (2019) pioneered the application of a U-net variant for the fully automatic segmentation of cerebral arteries. They proposed a “half U-net,” which reduced the number of channels in each layer by half compared to the original design. This simplified model demonstrated superior performance to traditional graph-cut algorithms, validating the U-net's efficacy for cerebrovascular MRA segmentation. However, the authors noted that the model was more effective for segmenting larger vessels than smaller ones. To further improve segmentation performance, Guo et al. (2021) developed a multi-view fusion approach termed M-u-net (multiple-u-net). This method involved training separate U-net models on axial, coronal, and sagittal views of TOF-MRA data. The features from these three orthogonal networks were then fused, followed by post-processing using connected component analysis. The resulting M-u-net model proved effective for the automated segmentation of cerebrovascular structures from TOF-MRA images. The major deep learning models for intracranial vessel segmentation are summarized in Table 1, and their key clinical translation implications are highlighted in Box 1.

**Table 1:** Comparison of major deep learning models for intracranial vessel segmentation.

Model	Core architectural features	Representative performance (dice similarity coefficient, DSC)	Key advantages	Key limitations	Clinical application scenarios	Key references
U-Net	Classic encoder-decoder architecture with skip connections.	DSC: 0.75–0.88 (on MRA/CTA data)	Simple and efficient structure, robust with small sample sizes; skip connections preserve spatial details	Requires extension to 3D U-Net for volumetric data; limited receptive field with shallow depth may miss small vessels	Fast and accurate segmentation of major cerebral vessels; suitable for routine clinical image quality	Ronneberger et al., 2015
DeepMedic	Parallel multi-scale convolutional pathways designed for 3D volumetric data	DSC: 0.82–0.90 (particularly robust for small vessels)	Native 3D data processing, effective multi-scale context capture; high accuracy for small vessel segmentation	Large number of parameters, high computational resource consumption; longer training times	Research scenarios requiring high-precision small vessel segmentation (e.g., lenticulostriate arteries); cerebral small vessel disease analysis	Kamnitsas et al., 2017
U-Net++	Introduces dense skip connections on U-Net basis for improved gradient flow	DSC: 0.80–0.92 (reports show superior performance to basic U-Net)	More effective fusion of feature maps at different depths, enhances boundary segmentation accuracy	More complex structure, increased computational and memory overhead	Applications requiring high-precision vessel boundary segmentation, e.g., geometric model generation for CFD simulations	Zhou et al., 2018
VesselNet	Variant designed specifically for vessel segmentation, often incorporating vascular centerlines or geometric priors	DSC: > 0.85 (in specific vascular tree segmentation tasks)	Optimized for tubular structure and topology of vessels, better maintains vascular continuity	Potentially less generalizable than purely data-driven models; dependent on quality of prior knowledge	Complete reconstruction of complex cerebrovascular trees (e.g., circle of Willis); vascular topology analysis	Tetteh et al., 2020

Box 1 **Key Points for Clinical Translation**

The accurate segmentation of intracranial vessels, particularly small deep perforators, is not just a technical exercise. It enables the precise measurement of stenosis length and the identification of subtle aneurysms or vascular malformations that are critical for diagnosis and pre-interventional planning, potentially leading to earlier intervention and improved patient outcomes.

## 6 Advances in deep learning for carotid artery segmentation across imaging modalities

In contrast to the extensive research on intracranial artery segmentation, deep learning applications dedicated to carotid artery imaging remain more limited. The quantification of carotid plaque burden is critical for assessing and monitoring the progression or regression of carotid atherosclerosis. Specifically, measuring the vessel wall volume (VWV) derived from segmented media-adventitia (MAB) and lumen-intima (LIB) boundaries serves as a key imaging biomarker for precisely monitoring plaque progression or regression. Zhou R. et al. (2019) employed both a custom CNN and a U-Net to segment carotid MAB and LIB boundaries from ultrasound images, proposing a semi-automatic, deep learning-based method for 3D ultrasound (3DUS). For MAB segmentation, the problem was framed as a pixel classification task. A dynamic CNN was designed to classify image patches sampled along normals to an initial contour, with the model being fine-tuned dynamically for each case. For LIB segmentation, a U-Net was trained end-to-end on regions of interest. The proposed carotid plaque segmentation method demonstrated high accuracy and reproducibility, facilitating reliable VWV measurement for clinical assessment. Tsakanikas et al. (2020) proposed a hybrid iterative framework for carotid vessel segmentation in multispectral MR images. The algorithm integrates a U-Net-based CNN with morphological active contours to achieve precise segmentation of both the lumen and outer wall. This approach enables the generation of accurate 3D vessel models, which are essential for improving computational fluid dynamics (CFD) simulations and for developing models of atherosclerotic plaque progression.

DeepMedic is an 11-layer, dual-path deep convolutional neural network originally designed for brain lesion segmentation. Ziegler et al. (2021) adapted this network for the multi-class segmentation of the common, internal, and external carotid arteries on contrast-enhanced magnetic resonance angiography (CE-MRA) images. Given that the carotid bifurcation is a prevalent site for atherosclerosis, its geometry—including branch diameters, bifurcation angles, and tortuosity—can serve as patient-specific risk markers (Spiliopoulos et al., 2024). The study aimed to perform precise segmentation to enable the quantification of these geometric descriptors. Several modifications were made to the original DeepMedic architecture (Ziegler et al., 2021; Bizjak and Špiclin, 2023). To capture multi-scale contextual information, an additional downsampling path was incorporated alongside the existing paths, utilizing subsampling factors of 3 and 5. Furthermore, kernel sizes were enlarged throughout the network to increase the receptive field. Specifically, the number of feature maps in deeper layers was reduced, the neuron count in two fully connected layers was increased, and the kernel shapes were altered from cubic to rectangular prisms to better match the anisotropic dimensions of typical MR image volumes and improve the segmentation of tubular vascular structures. However, the method required manual initialization in the form of bounding boxes around the left and right carotid regions, along with subsequent post-processing, which may have impacted the fully automated performance and the final segmentation accuracy. Nonetheless, the work established a pipeline for deriving quantitative geometric risk markers from carotid CE-MRA.

Vessel segmentation in computed tomography angiography (CTA) presents distinct challenges compared to magnetic resonance angiography (MRA). While extensive research on CTA segmentation exists for coronary arteries, the application to carotid arteries—which exhibit different atherosclerotic pathophysiology—has received comparatively less attention, particularly with deep learning methods. Traditional approaches for carotid CTA segmentation and quantification can be categorized into three groups: vessel model-based methods, image feature-based techniques, and extraction scheme-driven approaches. Hemmati et al. (2015) proposed a semi-automatic carotid lumen segmentation method bridging elements of the first and third categories. Their technique employed a 3D Hessian-based extraction scheme to obtain vessel centerlines, followed by model-driven segmentation based on these centerlines. The three-stage pipeline included: (1) preprocessing with mean shift filtering to enhance regional homogeneity while preserving edges; (2) centerline extraction for the common, internal, and external carotid arteries using a shortest-path algorithm based on multiscale Hessian analysis, with refinement to exclude very small branches; and (3) 3D level set segmentation to handle complex geometries.

This design addresses several inherent challenges in carotid CTA segmentation: the large number of slices, intensity variations across slices, the presence of adjacent tissues and veins, and non-circular lumen shapes at stenoses and bifurcations. The incorporation of a centerline extraction step is common to improve robustness. A particularly difficult task is the accurate segmentation of the arterial lumen in the presence of heavily calcified plaques. The method by Hemmati et al. showed agreement with manual segmentation, demonstrating capability in segmenting small branches. However, a fundamental limitation arises in scenarios of complete arterial occlusion, where the prerequisite centerline extraction fails, leading to segmentation breakdown. This limitation, shared by other manual or semi-automatic methods, underscores the need for more robust, fully automatic segmentation networks in carotid CTA analysis.

Vessel segmentation in carotid computed tomography angiography (CTA) is particularly challenging due to several factors. First, the high intensity of surrounding bony structures (e.g., skull base) can cause adhesion and extraction of non-vascular tissues, interfering with accurate diagnosis. Second, the direct processing of entire high-resolution 3D CTA volumes is computationally prohibitive, while downsampling sacrifices detail and naive patch sampling leads to severe class imbalance, as vessels constitute a minuscule fraction of the total volume. Consequently, the field grapples with two primary obstacles: the need for fully automated techniques that obviate manual region-of-interest delineation, and effective strategies to handle extreme data imbalance. Promising directions include coarse-to-fine (C2F) segmentation pipelines and the use of multi-planar analysis. Notable architectures for this task include 3D U-Net, its variants like RA U-Net (incorporating residual and attention modules) and Isensee U-Net (with deep supervision), and state-of-the-art multi-planar U-Nets.

Addressing these challenges, Wang et al. (2021) proposed a C2F pipeline with a multi-planar D-SEA U-Net for fully automatic carotid segmentation. The pipeline first uses a coarse segmentation module to locate candidate carotid regions from the entire 3D volume. Subsequently, a fine segmentation module processes these regions at full resolution to obtain precise boundaries. The D-SEA U-Net itself is a multi-planar 2D network that processes orthogonal views. When evaluated within this C2F framework, it achieved the best Dice Similarity Coefficient (DSC), sensitivity, AUC, and average surface distance compared to classic 3D U-Net variants. A key finding was that multi-planar 2D segmentation networks (planar U-Net and multi-planar D-SEA U-Net) significantly outperformed their 3D counterparts in this task. The multi-planar D-SEA U-Net exhibited the lowest variance in performance, demonstrating superior robustness and optimal preservation of vessel integrity and continuity.

In a complementary approach, Fu et al. (2020) developed “CerebralDoc,” a system for reconstructing head and neck vasculature (aorta, carotid, and intracranial arteries) from CTA. Their system is based on an optimized U-Net architecture informed by physiological anatomy. The model comprises two main components: a ResU-Net for initial vessel extraction and bone stripping, and a Connected Growth Prediction Model (CGPM) to ensure topological integrity and correct for missing segments. This method processes the original 3D slices and uses an automated strategy for model parameter optimization. In clinical evaluation, volume rendering (VR) and other reconstructions based on CerebralDoc's output displayed more distal cerebrovascular branches and clearer vessel images than manual reconstruction, proving particularly beneficial for assessing ischemic regions and collateral circulation. The system learns vessel-specific features rather than relying on simple thresholds, enabling accurate signal capture. However, its application is currently confined to head and neck CTA, and like many deep learning models, it relies on large-scale training data, posing a limitation for small-sample scenarios.

Chen et al. (2021) proposed a multi-scale deep network (MDNet Vb) for vascular segmentation, which incorporates a dual-path, multi-resolution architecture and a novel loss function to enhance efficiency and accuracy. The network comprises two parallel streams: a normal-resolution path using a 3D U-Net to capture local vessel details with lower memory footprint, and a low-resolution path using a 3D fully convolutional network (FCN) on downsampled inputs to learn global contextual and larger-scale morphological features. In parallel, to address the prevalent class imbalance between vessel and background voxels, the authors introduced a voxel-level class-balanced loss function, which reweights the contribution of each class based on its effective sample frequency. Evaluated on cardiac CTA and cerebral MRA datasets, MDNet Vb outperformed other state-of-the-art methods, achieving high accuracy with reduced parameter count. While this demonstrates its strong potential for general vascular image segmentation, its specific efficacy for supra-aortic arteries (e.g., carotid and vertebral arteries) remains to be validated.

In a related but distinct domain, Radl et al. (2022) focused on segmenting the aortic vessel tree—from the ascending aorta to the iliac arteries—from whole-body CTA scans. The study employed a semi-automated, editor-guided workflow using 3D Slicer, involving preprocessing (e.g., gradient anisotropic diffusion filtering), local thresholding, and manual correction for artifacts. This approach successfully generated aortic tree models but did not perform detailed segmentation of individual supra-aortic branches (e.g., carotid, subclavian). Consequently, while the pipeline provides a foundation for large-vessel segmentation, it is not directly applicable to the automated, fine-grained segmentation of supra-aortic arteries required for cerebrovascular assessment. Future integration of dedicated supra-aortic artery CTA datasets could potentially adapt this framework for that purpose. The major deep learning models for carotid artery segmentation and plaque analysis are summarized in Table 2, and their clinical translation implications are highlighted in Box 2.

**Table 2:** Comparison of major deep learning models for carotid artery segmentation and plaque analysis.

Model	Core architectural features	Representative task and performance	Key advantages	Key limitations	Clinical application scenarios	Key references
3D U-Net	3D extension of standard U-Net, handles anisotropic images	Lumen/wall segmentation DSC: 0.85–0.93	Leverages 3D spatial context effectively; accurate for carotid plaque volume quantification	May require special preprocessing or architectural adjustments for high-resolution anisotropic MRI data	Automated quantification of carotid plaque burden; monitoring volume changes in longitudinal studies	Çiçek et al., 2016
nnU-Net	Framework that self-configures U-Net parameters, not a fixed architecture	Plaque component segmentation (e.g., lipid-rich necrotic core) DSC: 0.75–0.82	High automation, no need for manual hyperparameter tuning; demonstrates excellent generalization across tasks	“Black-box” design, its automatic configuration logic is not easily controlled or understood by researchers	Rapid, robust baseline segmentation of vessel wall and plaque components from multi-center, multi-sequence MRI data	Isensee et al., 2021
Transformer-based models	Utilizes self-attention mechanisms to capture long-range dependencies	Plaque component classification accuracy: >90%	Strong global contextual modeling, beneficial for distinguishing plaque components with similar textures (e.g., fibrous tissue vs. calcification)	Requires large datasets for training, prone to overfitting; high computational complexity, relatively slow inference	High-precision identification of vulnerable plaque features (e.g., fibrous cap rupture, intraplaque hemorrhage) for stroke risk stratification	Cao et al., 2023
Recurrent convolutional network (RCNN)	Combines CNNs with recurrent units to process sequential slices	Vessel centerline extraction average distance error: < 0.5 mm	Effectively utilizes continuity between adjacent slices, produces smooth and accurate vessel centerlines	Primarily for centerline/contour extraction, limited capability for volumetric segmentation of plaque components	Providing precise vascular geometric models for CFD simulations or lumen stenosis degree calculation	Zheng et al., 2022

Box 2 **Key Points for Clinical Translation**

The ability to automatically quantify carotid plaque volume and characterize its composition (e.g., identifying lipid-rich necrotic cores) moves beyond simple stenosis measurement. This provides a more powerful, imaging-based biomarker for individualized stroke risk assessment, which could guide decisions regarding aggressive medical therapy or surgical intervention.

## 7 Beyond segmentation: deep learning in hemodynamics and vessel wall analysis

While vascular segmentation provides crucial anatomical insights, the pathophysiology of cerebrovascular diseases like stroke and headache disorders is deeply rooted in dynamic hemodynamic alterations and structural vessel wall pathology. Deep learning is now powerfully addressing these domains, moving beyond static anatomy to functional and tissue-level characterization (Lin et al., 2025).

In hemodynamic analysis, DL has revolutionized the computation of intravascular flow parameters. Traditional computational fluid dynamics (CFD) applied to patient-specific models derived from angiography is computationally intensive and time-consuming, hindering clinical translation (Sankaran et al., 2016). DL models, particularly those based on convolutional and physics-informed neural networks, can now estimate key hemodynamic metrics, such as wall shear stress and pressure gradients, directly from medical images in near real-time (Yang X. et al., 2025). For instance, recent studies have demonstrated that DL-based surrogates can accurately compute fractional flow reserve in intracranial arteries, identifying hemodynamically significant lesions that predispose to ischemic stroke (Qi et al., 2025). Furthermore, these models are uncovering subtle flow patterns in the posterior circulation that may be linked to altered cerebral perfusion in certain types of migraine, offering a potential mechanistic explanation for headache symptoms (Morelli et al., 2025).

Concurrently, DL has dramatically advanced the analysis of the vessel wall itself using high-resolution magnetic resonance imaging (HR-MRI). The identification of plaque composition—such as lipid-rich necrotic core, intraplaque hemorrhage, and fibrous cap integrity—is critical for assessing stroke risk, especially in carotid disease (Donners et al., 2021). CNNs and vision transformers are now achieving expert-level performance in automatically segmenting these components and quantifying morphological features like plaque burden and stenosis (Lin et al., 2023). More importantly, DL can identify imaging signatures of inflammation, a key driver of plaque vulnerability. By analyzing subtle signal intensity patterns and texture features on post-contrast HR-MRI, deep learning models can infer inflammatory activity within the plaque, potentially serving as a non-invasive biomarker for monitoring disease activity and response to therapy (Klüner et al., 2024). This is highly relevant to headache disorders, as evolving research suggests that neurovascular inflammation may be a shared pathway in the pathogenesis of both migraine and stroke (Anwar et al., 2025).

In summary, the application of deep learning to hemodynamics and vessel wall characterization represents a paradigm shift from describing vascular anatomy to understanding dynamic pathophysiology. These advanced analyses provide a more comprehensive assessment of stroke risk and offer novel insights into the neurovascular mechanisms underlying headache disorders, paving the way for personalized management strategies and a deeper understanding of cerebrovascular health. The key clinical translation implications of deep learning-based hemodynamic and vessel wall analysis are highlighted in Box 3.

Box 3 **Key Points for Clinical Translation**

The ability to compute hemodynamic forces like wall shear stress *near real-time* can transform the assessment of stroke risk from a static anatomical measurement to a dynamic functional one, potentially identifying high-risk lesions faster than traditional simulations. Similarly, automatically characterizing plaque composition and inflammation from MRI provides a powerful, non-invasive tool for personalizing stroke prevention strategies and monitoring treatment response, offering new insights into the vascular contributions to headache disorders.

## 8 Innovative paradigms for addressing data challenges

Beyond the established approach of building larger multi-center databases, several emerging learning paradigms offer innovative pathways to address data heterogeneity and scarcity, which is particularly relevant for niche domains like headache neuroimaging where large, labeled datasets are rare. Federated learning presents a groundbreaking framework for training models across multiple institutions without sharing raw patient data, thus preserving privacy and mitigating legal hurdles. In this setup, a global model is collaboratively trained by aggregating model updates (e.g., gradients) from local models trained on their respective private datasets. This is ideally suited for pooling data from different hospitals to build a robust model for, for instance, classifying migraine-related brain changes, while keeping the sensitive imaging data decentralized.

Furthermore, self-supervised learning (SSL) provides a powerful method to leverage the vast amounts of unlabeled imaging data that are readily available in clinical archives. SSL models are pre-trained on pretext tasks—such as predicting missing parts of an image or solving jigsaw puzzles—using only unlabeled data, thereby learning rich and generalizable representations of the data manifold. This pre-trained model can then be fine-tuned for specific downstream tasks (e.g., segmenting the trigeminal nerve or classifying aura patterns) with a relatively small amount of labeled data, effectively overcoming the label-scarcity bottleneck.

Finally, generative AI, particularly Generative Adversarial Networks (GANs) and Diffusion Models, can be harnessed to create high-quality, synthetic neuroimaging data. These models can generate anatomically plausible and label-accurate synthetic images to augment small training datasets, improving model robustness and generalization. More importantly, they can be used to simulate rare pathological presentations or to create balanced datasets, thereby mitigating class imbalance issues that are common in clinical data. Together, federated learning, self-supervised learning, and generative AI represent a paradigm shift from merely collecting more data to intelligently and ethically leveraging existing data resources to advance AI in cerebrovascular and headache research.

## 9 Technical limitations and bottlenecks

Despite the remarkable progress, several inherent technical limitations of current DL models impede their seamless clinical integration. A primary concern is data dependency and generalization. Models trained on datasets from specific scanner vendors, protocols, or patient populations often experience significant performance degradation when applied to external data, a phenomenon known as domain shift. This underscores the acute need for large-scale, multi-center, and multi-vendor datasets for robust training. Secondly, the “black box” nature of complex DL models remains a critical barrier. The lack of interpretability and explainability hinders clinical trust and adoption, as physicians are understandably reluctant to base critical decisions on opaque algorithmic outputs. Developing techniques like saliency maps or uncertainty quantification is paramount for verifying model reasoning and identifying potential failure modes. Thirdly, the effective integration of hemodynamic parameters (e.g., fractional flow reserve, wall shear stress) derived from anatomical segmentations into a unified diagnostic framework is still in its infancy. Most current studies focus solely on morphological analysis, whereas the next frontier lies in creating end-to-end systems that can seamlessly translate segmented geometries into clinically actionable hemodynamic insights. Finally, Although U-Net demonstrates excellent performance on clean images, its segmentation continuity is susceptible to breakdown in the presence of motion artifacts in low-dose CTA, which are frequent in emergency settings. This degradation can result in an inaccurate estimation of stenosis length. To address this limitation, recent research has incorporated adversarial training or motion-correction modules into the learning framework.

## 10 Ethical and societal considerations

The translation of deep learning tools from research to clinical practice necessitates a rigorous examination of associated ethical and societal implications, which are as critical as their technical performance. Firstly, the development of robust models hinges on large-scale, multi-center datasets, raising paramount concerns regarding patient data privacy and security. Compliance with regulations such as the General Data Protection Regulation (GDPR) and the Health Insurance Portability and Accountability Act (HIPAA) is a baseline requirement. However, ethical stewardship extends beyond mere compliance. Techniques like federated learning, which trains models across institutions without sharing raw patient data, present a promising paradigm for preserving privacy while leveraging diverse datasets. Secondly, the issue of algorithmic bias demands urgent attention to prevent the perpetuation or exacerbation of healthcare disparities. If DL models are trained on non-representative datasets—for instance, cohorts lacking diversity in ethnicity, socioeconomic status, or geographic location—their performance may degrade significantly when applied to underrepresented populations. This could lead to inaccurate diagnoses for minority groups, thereby worsening existing inequalities in cerebrovascular care, such as the delayed diagnosis of certain headache disorders or strokes. Therefore, proactive efforts to curate diverse and inclusive training datasets are imperative to develop fair and generalizable algorithms.

Finally, the “black-box” nature of many complex DL models poses a significant challenge to their clinical adoption. The lack of transparency and interpretability can erode trust among clinicians, who are ultimately responsible for diagnostic and therapeutic decisions. Without understanding the rationale behind a model's output—for instance, which image features led to the classification of a plaque as vulnerable—physicians may be hesitant to integrate these tools into critical decision-making pathways. Advancing Explainable AI (XAI) techniques is thus a key research direction. Methods such as saliency maps, which highlight the image regions most influential to the model's decision, can provide crucial insights and foster trust. Furthermore, establishing accountability frameworks is essential for clinical deployment. In conclusion, addressing these ethical challenges—data privacy, algorithmic bias, and model transparency—is not an ancillary concern but a prerequisite for the responsible development of equitable, accountable, and trustworthy AI tools in cerebrovascular medicine.

## 11 Conclusion

This review has systematically examined the progress and applications of artificial intelligence, particularly deep learning (DL), in the imaging-based diagnosis and evaluation of cerebrovascular diseases. From the evolution of core network architectures to their deployment across intracranial, carotid, and coronary vascular territories, the evidence indicates that DL has evolved from a conceptual promise to a transformative tool reshaping the paradigm of stroke diagnosis and management. By automating and standardizing tasks such as vessel segmentation, plaque quantification, stenosis grading, and hemodynamic assessment, DL models—exemplified by architectures such as U-Net, DeepMedic, and their variants—have demonstrated the potential to achieve diagnostic accuracy and efficiency comparable to, or even exceeding, expert manual analysis. This offers a tangible pathway to address critical clinical bottlenecks, including interpreter variability, excessive workload, and diagnostic delays.

However, translating this technical potential into robust, widespread clinical practice faces several pivotal challenges. First, generalizability remains constrained by data heterogeneity and scarcity. Model robustness is heavily dependent on large-scale, high-quality, and meticulously curated annotated datasets from multiple centers and scanner platforms, a requirement not yet met by most single-center studies. Second, a critical gap persists in rigorous, prospective clinical validation. Many algorithms have not been subjected to such trials, and the absence of standardized benchmarks hampers objective performance comparison and the establishment of clinical credibility. Third, the integration of AI tools into established clinical workflows presents a significant hurdle. Achieving seamless integration within existing Picture Archiving and Communication Systems and clinical decision-making protocols—moving from proof-of-concept to routine practice—requires concerted interdisciplinary efforts and must demonstrate a clear improvement in workflow efficiency or patient outcomes.

To advance the field toward robust clinical integration, future efforts should prioritize several key directions. First, a focused effort toward creating standardized, open-access, multi-center, and multimodal imaging databases alongside algorithm validation platforms is needed, enabling fair benchmarking and enhancing model generalizability. Second, advancing Explainable AI (XAI) is paramount to render model decision-making processes interpretable and transparent for clinicians, thereby building essential trust and facilitating effective human-AI collaboration. Third, developing models capable of multi-task learning and true multimodal fusion—integrating data from CTA, MRA, ultrasound, and electronic health records—could enable a paradigm shift from analyzing isolated imaging features to comprehensive, patient-level risk stratification. Fourth, and most fundamentally, fostering deep, prospective, and interdisciplinary collaboration among academia, industry, and clinical medicine must be at the core of this endeavor. Clinicians must be engaged from the outset to define clinically meaningful endpoints, while engineers and data scientists must gain a nuanced understanding of real-world clinical constraints and workflows. Finally, Future efforts must actively integrate the expertise of not only clinicians and computer scientists but also specialized neurologists with deep understanding of headache pathophysiology, biomedical engineers for device and algorithm co-design, and biostatisticians for robust phenotypic definition. Establishing practical frameworks, such as large-scale, curated, and shared headache neuroimaging repositories with standardized protocols, will be crucial to fuel innovation. Such collaborative ecosystems are essential for developing clinically relevant, generalizable, and ethically deployed AI tools that can ultimately improve patient outcomes in cerebrovascular and headache disorders.

In conclusion, the application of deep learning in cerebrovascular image analysis is ushering in a new era of stroke care, characterized by greater precision, efficiency, and accessibility. Its ultimate objective is not to replace the clinician, but to function as an augmented intelligence (AI) partner. By automating the quantitative analysis of vast datasets and mitigating subjective interpretive variability, it empowers clinicians to transition their role from one of labor-intensive, subjective measurements to one of efficient patient evaluation and personalized therapeutic decision-making. Realizing this vision hinges on sustained technological innovation, rigorous clinical validation, and deepened interdisciplinary collaboration.
